# Targeting Leukemia Stem Cells in the Bone Marrow Niche

**DOI:** 10.3390/biomedicines6010022

**Published:** 2018-02-21

**Authors:** Sarah K. Tasian, Martin Bornhäuser, Sergio Rutella

**Affiliations:** 1 Division of Oncology and Center for Childhood Cancer Research, Children’s Hospital of Philadelphia, Philadelphia, PA 19104, USA; tasians@email.chop.edu; 2 Department of Internal Medicine I, University Hospital Carl Gustav Carus, Technische Universität Dresden, 01069 Dresden, Germany; martin.bornhaeuser@uniklinikum-dresden.de; 3 John van Geest Cancer Research Centre, Nottingham Trent University, Nottingham NG11 8NS, UK

**Keywords:** leukemia stem cell, microenvironment, immune biomarker, immunotherapy, immune checkpoints, bone marrow, prognosis

## Abstract

The bone marrow (BM) niche encompasses multiple cells of mesenchymal and hematopoietic origin and represents a unique microenvironment that is poised to maintain hematopoietic stem cells. In addition to its role as a primary lymphoid organ through the support of lymphoid development, the BM hosts various mature lymphoid cell types, including naïve T cells, memory T cells and plasma cells, as well as mature myeloid elements such as monocyte/macrophages and neutrophils, all of which are crucially important to control leukemia initiation and progression. The BM niche provides an attractive milieu for tumor cell colonization given its ability to provide signals which accelerate tumor cell proliferation and facilitate tumor cell survival. Cancer stem cells (CSCs) share phenotypic and functional features with normal counterparts from the tissue of origin of the tumor and can self-renew, differentiate and initiate tumor formation. CSCs possess a distinct immunological profile compared with the bulk population of tumor cells and have evolved complex strategies to suppress immune responses through multiple mechanisms, including the release of soluble factors and the over-expression of molecules implicated in cancer immune evasion. This chapter discusses the latest advancements in understanding of the immunological BM niche and highlights current and future immunotherapeutic strategies to target leukemia CSCs and overcome therapeutic resistance in the clinic.

## 1. Introduction

**Figure 1:**
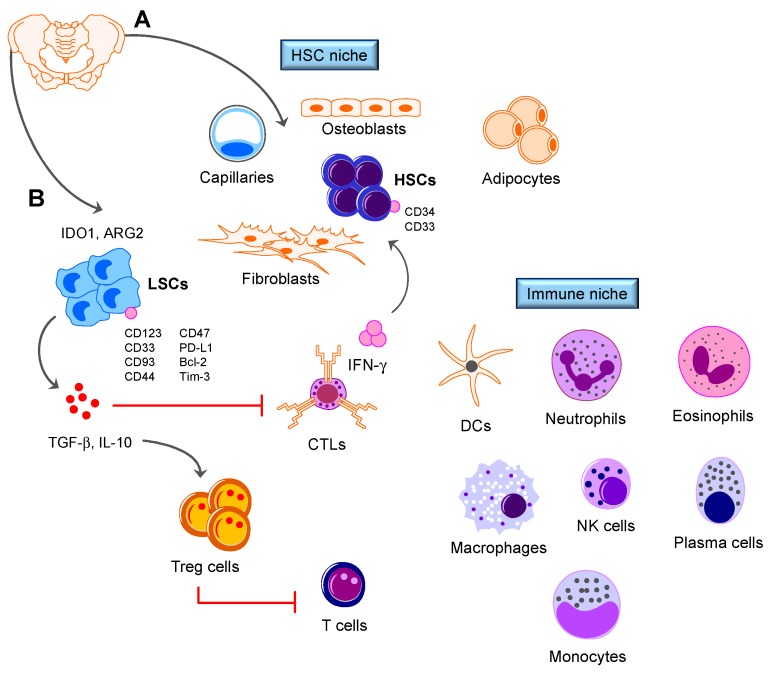
The immune landscape of normal (**A**) and leukemic (**B**) BM. The BM microenvironment hosts a variety of immune cell types, including T cells, B cells, plasma cells, dendritic cells, neutrophils, macrophages, eosinophils and regulatory T cells. Immune cells support steady-state and emergency hematopoiesis, provide an immune privileged niche that protects HSCs from immune destruction and contribute to leukemia control. Candidate leukemia stem cell markers, including CD123 [5], CD44 [6], Bcl-2 [7] and Tim-3 [8], as well as markers of normal hematopoietic stem cells are shown. Microenvironmental soluble factors, such as interferon (IFN)-γ produced by cytotoxic T cells, might promote leukemia cell proliferation [9]. IDO1 = Indoleamine 2,3-dioxygenase-1; ARG2 = arginase-2; LSC = leukemia stem cell; HSC = hematopoietic stem cell; DC = dendritic cell; CTL = cytotoxic T lymphocyte. Red lines denote feedback inhibition.

The BM niche is a 3D structure situated in close proximity to trabecular bone [1]. The cellular components of the BM niche can be categorized into two functional types: (1) essential cells such as endothelial cells, mesenchymal stromal cells (MSCs), and megakaryocytes, which provide close proximity signals to hematopoietic stem cells (HSCs) that are destined for differentiation and subsequent export into the circulation, and (2) accessory cell types such as osteoblasts, specialized tissue-resident macrophages, and nerve cells, which exert long-range and often indirect influences on HSCs [2]. HSCs reside within specialized areas of the BM microenvironment, defined as two distinct osteoblastic and vascular niches (Figure 1) [3]. The osteoblastic niche, localized at the inner surface of the bone cavity, provides a microenvironment for long-term HSCs which contribute to hematopoiesis as quiescent or slow-cycling cells. The vascular niche consists of sinusoidal endothelial cells lining blood vessels, and promotes proliferation and differentiation of actively cycling, short-term HSCs [3].

Several other cellular elements with specialized functions, including immune cells, provide distinct chemical signals and physical interactions essential for HSC maintenance and regulation of blood production [2]. The niche also encompasses matrix elements and micro-vessels which shape the unique biochemical composition of the BM milieu. For instance, quiescent HSCs tend to reside in poorly perfused, relatively hypoxic areas which trigger metabolic adaptations that prevent differentiation [4].

The cancer stem cell (CSC) hypothesis was first described in leukemia in 1994 and stipulates that cancer develops in a hierarchical manner from CSCs that self-renew and give rise to a differentiated cell progeny by asymmetric division [10,11,12,13]. Leukemia stem cells (LSCs) have been phenotypically and functionally characterized in chronic myeloid leukemia (CML) [14], acute myeloid leukemia (AML) and myelodysplastic syndromes (MDSs) [15]. LSCs rely on a number of signaling pathways that are associated with the ability to self-renew and that are shared with normal HSCs, such as Wingless-type MMTV integration site family member (WNT)/β-catenin, Hedgehog and B-cell-specific Moloney murine leukemia virus integration site-1 (BMI-1) [16,17], and reside in a quiescent state, a property which influences their relative sensitivity to chemotherapeutic drugs targeting cycling cells [3]. The interaction between CXCR4 on leukemia progenitors and CXCL12 on BM stromal cells favors LSC homing to the BM microenvironment [18]. Stromal-derived factor (SDF)-1α, a chemokine ligand for CXCR4 which is also produced by immature osteoblasts lining the endosteum region, induces significant calcium fluxes in blast cells from myelomonocytic AMLs [19]. In contrast, promyelocytic AMLs, as well as M4 AMLs with eosinophilia, displayed intermediate activity. Accordingly, CXCR4 expression was low in undifferentiated AML, myeloid AML and erythroid AML, but high in promyelocytic AML, myelomonocytic AML and B-lineage acute lymphoblastic leukemia (ALL). CXCR4 expression is significantly higher in AML patients with Fms-like tyrosine kinase-3 (FLT3) internal tandem duplication (ITD) [20]. Importantly, AML patients with a high CXCR-4 expression in CD34^+^ cells have a median relapse-free survival (RFS) of only 8.3 months [20]. The weekly administration of anti-human CXCR4 antibodies to mice previously engrafted with primary AML blasts decreases the number of AML cells in the blood, BM and spleen, with minimal effects of the number of normal HSCs engrafted into NOD/SCID mice [21].

CML is a rare clonal disorder of HSCs associated with t(9;22) resulting in *BCR-ABL1* rearrangement and has an annual incidence of 1 to 2 cases per 100,000 individuals [22]. CML presents in chronic phase in 85–90% of patients and, if untreated, usually progresses to myeloid or lymphoid blast crisis within 5 years. Overall survival (OS) of patients with CML has dramatically improved with use of breakpoint cluster region/Abelson (BCR-ABL)1 fusion protein-targeting tyrosine kinase inhibitors (TKIs), such as imatinib or dasatinib, along with allogeneic hematopoietic stem cell transplantation (HSCT), with life expectancy in patients with CML approaching that of the general population [23]. However, the persistence of LSCs in CML remains an obstacle to cure in all patients [14]. CML becomes increasingly refractory to TKIs during progression to blast crisis. Mutations in the kinase domain (KD) of *BCR-ABL* are the most prevalent mechanism of acquired imatinib resistance [24]. CML LSCs with a CD34^+^CD38^−^ phenotype have been shown to express CD26, a cytokine-targeting surface enzyme that is not detectable on normal stem cells or LSCs in other hematological malignancies [25,26]. In functional assays, CD26 disrupted the SDF-1α-CXCR4 axis by cleaving SDF-1α and facilitated leukemia escape from the BM niche. Importantly, CD26^+^ LSCs decreased to low or undetectable levels after successful treatment with imatinib. The ability of CD26-expressing LSCs to engraft in mice was significantly reduced after their in vitro pre-treatment with gliptins. Intriguingly, 2 patients with CML receiving gliptins for concomitant diabetes mellitus had a decrease of BCR/ABL1 transcript levels during treatment. Patient-derived CML cells and LSCs in mouse models of CML express programmed death ligand-1 (PD-L1), the blockade of which triggers the loss of LSCs and prevents development of CML-like disease, if combined with T-cell immunotherapy [22,27]. CML LSCs could evade immune surveillance through a variety of molecular mechanisms, including the cytokine-mediated down-regulation of major histocompatibility complex (MHC) class II molecules [28].

Acute myeloid leukemia (AML) is the most common leukemia occurring in adults and the second most common leukemia of childhood. AML is genetically heterogeneous and is characterized by BM infiltration with abnormally differentiated and proliferating cells of hematopoietic origin. Current standard of care includes treatment with several cycles of high-dose chemotherapy and often includes allogeneic HSCT for patients with high-risk features such as adverse molecular or cytogenetic aberrations. Molecularly-targeted agents, such as midostaurin for FLT3^+^ patients and enasidenib for patients with isocitrate dehydrogenase-2 (IDH2) mutations, have been approved by the U.S. Food and Drug Administration in 2017 for use in patients with relapsed/refractory AML. Despite consolidation with HSCT for patients with high-risk AML, relapse-free and overall survival remains poor [29,30,31,32,33]. Cure is achieved in 35 to 40% of adult patients who are 60 years of age or younger and in 5 to 15% of patients who are older than 60 years of age [34]. The outcome in older patients who are unfit for intensive chemotherapy remains dismal with a median survival of 5 to 10 months. New therapeutic approaches are compulsory to improve outcomes.

The CSC model has been demonstrated in AML via cell sorting of multiple populations from 16 primary human AML samples and subsequent identification of LSC-containing fractions in murine xenotransplantation studies [35]. Analysis of gene expression from functionally validated populations demonstrated LSC-specific and HSC gene signatures and identified core transcriptional programs shared by LSCs and HSCs. Interestingly, both stem cell programs significantly and independently predicted patient survival.

The MDSs comprise a heterogeneous group of malignant HSC disorders that are characterized by a variable risk of transformation to AML [36]. The International Prognostic Scoring System (IPSS) allows MDSs to be divided into lower and higher risk categories, the latter being associated with higher blast counts, increased risk of leukemic transformation, and shorter median OS [37]. Cytokine dysregulation contributes to immune dysfunction in patients with MDS [38]. IL-32, which is constitutively expressed at high levels by stromal cells from patients with untreated MDS, impairs NK function and promotes apoptosis, which recapitulates inefficient hematopoiesis, a pathophysiological hallmark of MDSs [38].

Seminal studies in the late 1990s first described the prevalence of LSCs in primary human AML specimens using limiting-dilution transplantation assays, reporting LSC frequencies varying over a 500-fold range (from 1 in 10,000 to 1 in 5 million) [39,40]. The quiescence of both normal stem cells and LSCs is critically determined by interactions with the HSC niche, including endothelial cells, perivascular cells, adipocytes, macrophages and cells of the adaptive immune system [41]. The majority of AML samples express cell surface CD34, and most studies of LSCs have focused on the CD34^+^CD38^−^ cell compartment, which has been associated with leukemia initiation and relapse [42]. However, transplantation studies have shown that LSCs are also present in at least one other subpopulation, usually the CD34^+^CD38^+^ fraction or sometimes the CD34^−^ fraction, as shown in half of AML cases with mutated *nucleophosmin-1* (*NPM1*), a molecular lesion usually associated with low CD34 expression [43]. Further immunophenotypic characterization of LSC populations with antibodies associated with primitive cell types, such as CD123, CD33, CD117, CD90, CD44, did not reveal any clear association between surface expression profile and a lower oxidative state, which is generally indicative of LSC quiescence [7]. Cycling LSC populations have also been identified in AML harboring *KMT2A* (*MLL*) gene rearrangement and are characterized by the expression of CD93 [44], a transmembrane C-type lectin with unknown function that can be detected early during B-cell maturation in the BM and is re-induced during the differentiation on plasmablasts and plasma cells [45]. In addition, functionally-defined LSCs have been identified in populations from relapsed AML samples that contained all permutations of CD34 and CD38 expression, suggesting that LSCs are dynamic and unstable and can diverge and evolve with acquisition of different phenotypes at relapse [15]. Finally, *FLT3*-ITD mutations have been detected in primitive human stem/progenitor cells with a CD34^+^CD38^−^ phenotype, including lymphoid-primed multipotent progenitors (LMPP)-like cells [46].

Transcriptional profiling of phenotypically-defined HSC subsets has shown that IL-1 receptor accessory protein (IL1RAP) is dysregulated in clonotypic stem and progenitor cells from patients with high-risk (-7/7q-) AML and is over-expressed on lineage^−^CD34^+^CD38^−^ HSCs from patients with high-risk MDSs [47]. Also, IL1RAP expression was independently associated with poor OS in 3 independent cohorts of patients with cytogenetically-normal AML, being an even stronger prognostic factor than *FLT3* mutation status. In contrast, IL1RAP could not be detected on HSCs from patients with low-risk MDSs. Mechanistically, down-regulation of IL1RAP inhibited the clonogenic capacity of AML cells and led to increased apoptosis [47]. The role played by IL1RAP in regulating responses to pro-inflammatory IL-1, IL-33, and IL-36, as well as in regulating mast-cell and T-cell activation [48,49,50,51], suggests that innate immune signaling pathways might contribute to the survival and aberrant growth of HSCs from patients with MDS. STAT3 might be hypo-methylated and over-expressed in CD34^+^ HSCs from patients with MDSs [47]. Importantly, treatment of MDS HSCs with cell-permeable STAT3 inhibitors translated into a reduction of in vitro colony formation. Single-cell transcriptomic approaches could unravel the heterogeneity and selective resistance of CSC populations to molecularly-targeted approaches. A novel protocol that integrates fluorescence-activated cell sorting, high-sensitivity single-cell mutation detection and single-cell RNA sequencing of the same single cell was applied to analyze more than 2000 HSCs from patients with CML and to characterize molecular signatures of LSC subpopulations, including BCR-ABL expression, in human CML samples from diagnosis through remission and disease progression [52]. Interestingly, poor responders to TKIs showed upregulation of TGF-β- and TNF-γ-pathway-associated genes that might be selectively targeted in CML-SCs, combined with a highly quiescent CML-SC signature. HSC populations specific for blast crisis transformation of CML were identified and characterized in a patient in chronic phase who subsequently developed blast crisis. This elegant approach supports that TKI-resistant CML-SCs are transcriptionally distinct from quiescent normal HSCs and might be broadly applicable to other tumor contexts to identify therapy-resistant CSC subpopulations.

Given their inherent resistance potential to a variety of therapeutic modalities, including radiotherapy, chemotherapy, immunotherapy and molecularly-targeted drugs such as TKIs, LSCs likely contribute to treatment failure and leukemia recurrence. A recent study from the Princess Margaret Cancer Centre Group in Toronto has tracked the complex evolutionary history of AML within individual patients from the early stages of pre-leukemic development to diagnosis and through progression to relapse [53]. Somatic variants predicted to have a damaging effect on the encoded protein (protein-damaging variants) were defined as pre-leukemic if present in T cells sorted from patient samples or leukemic if absent in these cells. Evidence of a pre-leukemic cell population was found in 10 out of 11 patients. Functional studies also established that LSCs are genetically diverse both at diagnosis and relapse, indicating continued branching evolution after leukemic transformation. Interestingly, the multi-lymphoid progenitor (MLP) fraction exhibited the closest relationship to the evolving LSC sub-clones of the diagnostic sample, suggesting that LSCs with an MLP phenotype could drive AML initiation. In some patients, the cellular origin of relapse was a rare population of cells with a primitive HSC-like phenotype, which are already present at diagnosis before initiation of therapy. These cases were termed as having a primitive relapse origin. In other patients, relapse originated from cells with a more committed immuno-phenotype (relapse origin-committed cases). One important implication of this observation is that chemotherapy does not induce mutations leading to emergence of new clones, but rather may select for pre-existing sub-clones that are already therapy-resistant [53]. In three patients, no relapse variants could be validated in any of the sorted cell populations from the diagnostic sample.

Other studies have identified a primary LSC gene signature in AML using in vivo murine PDX models [54]. In approximately 50% of primary AML specimens, LSCs over-expressed CD32, CD25, or both antigens. A detailed analysis of CD32 expression levels revealed three distinct patterns, i.e., AML cases with a single positive peak (category CD32-a), CD32-negative AML cases with a bimodal expression and a minor but separable CD32^+^ population (category CD32-b) and CD32-negative AML cases with a single negative peak (category CD32-c) [54]. CD34^+^CD38^−^CD32^+^ and CD34^+^CD38^−^CD25^+^ LSCs could initiate AML development in NSG mice, were cell cycle-quiescent and chemotherapy-resistant in vivo and expressed the transcription factor *WT1* and the kinase *HCK*. CD32 and CD25 could thus represent valuable targets for LSC-directed therapy, as suggested by the maintenance of long-term multi-lineage hematopoietic reconstitution capacity by normal human HSCs depleted of CD32/CD25-expressing cells. However, CD32 was detected with high levels of expression in a large number of alveolar macrophages in the lung [54]. Interestingly, AML patients with CD25 expression greater than 10% reportedly have higher minimal residual disease frequency after the first cycle of induction chemotherapy and a significantly shorter OS and RFS compared with patients showing a lower expression of CD25 [55]. In multivariate analysis, CD25 expression was an independent adverse factor for OS and RFS, especially when combined with *FLT3-*ITD positivity.

A list of differentially expressed genes in CD34^+^CD38^−^ LSCs versus their CD34^+^CD38^+^ non-LSC counterparts was recently generated using BM samples from 78 patients with AML [56]. The prognostic accuracy of this 17-gene signature (“LSC17 score”) was suggested by its correlation with higher percentages of BM blast cells at diagnosis, higher incidence of *FLT3*-ITD mutation and adverse cytogenetic features, higher relapse rates and lower response rates to induction chemotherapy. Furthermore, a high LSC17 score was associated with shorter OS irrespective of whether or not patients received a subsequent allogeneic stem cell transplantation.

The LCS17 score could also support the identification of low-molecular risk (LMR) AML cases at diagnosis, as shown in a large training cohort comprising patients of all subtypes, including 9% LMR cases, i.e., patients with normal cytogenetics, *NPM1* mutation and no *FLT3-*ITD [57]. A 13-gene sub-score accurately identified LMR patients in six large independent validation cohorts sparring approximately 1600 patients with cytogenetically and molecularly diverse AML [57]. This strategy could enable the evaluation of novel upfront treatment strategies for higher-risk LMR cases. The LSC17 gene signature was also shown to predict clinical outcome in pediatric AML [58]. Patients in the high-LSC17 cohort had a higher incidence of adverse cytogenetics and recurrent mutations, such as *FLT3*-ITD, and conversely, the low-scoring group had a higher percentage of favorable cytogenetics. In multivariate analysis, the LSC17 score was a strong independent prognostic indicator [58].

## 2. The Bone Marrow (BM) Immune Microenvironment

The BM is conventionally viewed as a primary lymphoid organ containing various immune cell populations (Figure 1). Billions of lymphocytes recirculate through the BM per day. Lymphocytes are distributed through the BM parenchyma and stroma, are condensed in follicle-like structures and encompass 8–20% of BM mononuclear cells, with a T-cell to B-cell ratio of 5:1. Antibody-producing plasma cells account for 1% of the BM mononuclear population. Plasma cells are found in close proximity to CXCL12-abundant reticular cells (RCs) and are dependent on CXCL12 signaling through CXCR4 for BM homing [59]. Other mature cell types, such as megakaryocytes and eosinophils, have been shown to contribute to the plasma cell niche [60].

Early in lymphoid development, B-cell precursors remain in the BM, while T-cell progenitors migrate to the thymus. RCs, a subpopulation of MSCs identified in a genetic mouse model [61], are detected in close association with pre-pro-B cells, the earliest B-cell precursors [62]. RCs also maintain HSCs in an undifferentiated state, as shown by accelerated myeloid differentiation in response to RC ablation [63]. Clusters of dendritic cells (DCs) co-localize with naïve T cells and B cells in the BM perisinusoidal space, as shown by multiphoton imaging [64]. BM-resident DCs deliver survival signals to recirculating B cells through the production of macrophage migration-inhibitory factor (MIF) and their conditional ablation leads to the specific loss of mature B cells [64]. The factors required for T-cell survival in the BM are less clearly defined. Perisinusoidal DCs can cross-present blood-borne antigens to BM-resident T cells, pointing to a protective role against pathogens [65]. 

Approximately one-third of BM CD4^+^ T cells are regulatory T (Treg) cells in humans, including memory or “activated” Treg cells, the trafficking of which is regulated by CXCL12 under homeostatic conditions [66]. Naïve T cells contribute 20% of BM-resident CD8^+^ T cells, with the largest subsets (∼30% each) being CD45RA^−^CCR7^+^ central memory T cells and CD45RA^−^CCR7^−^ effector memory T cells [67]. A smaller fraction is comprised of CD45RA^+^CCR7^−^ effector T cells. Long-lived memory CD4^+^ T cells are localized in close contact with IL-7-secreting stromal cells [68]. IL-7 is responsible for maintaining T-cell quiescence in the absence of antigen receptor engagement and signaling. Experiments in mice have shown that central memory T cells adhere to BM micro-vessels more efficiently than effector T cells [67]. This interaction is mediated by P-selectin glycoprotein ligand (PSGL)-1 on circulating central memory T cells and selectins on endothelial cells. In addition, the α4 integrin VLA-4 and its vascular ligand VCAM-1 play a major role in central memory T-cell arrest in BM micro-vessels [67]. Interestingly, markers indicative of antigen experience, such as CD44 and CD122, can be detected on two-thirds of BM T cells.

Finally, myeloid immune cells, such as neutrophils and monocytes, reside in specific niches within the BM. Under steady-state conditions, both cell types express CXCR4 and are retained into the BM through CXCL12-induced signaling [69]. During inflammation, neutrophils are released through interaction with CXCL1 and CXCL2, i.e., CXCR2 ligands produced by megakaryocytes [70]. In contrast, monocytes are released through interaction of CCR2 with CCL2 produced by RCs, MSCs and endothelial cells [71].

The BM also serves other functions, acting as a secondary lymphoid organ where T-cell and B-cell responses are initiated. Other features of a secondary lymphoid organ include the presence of follicle-like structures, and the ability of the BM to control systemic diseases, such as inflammatory, infectious and autoimmune conditions. In mice, the BM contains 1–5% CD3^+^ T cells and 1–2% CD11c^+^ DCs in different stages of maturation and harbors DCs that capture, process and present antigens to naïve CD4^+^ and CD8^+^ T cells, as revealed by the formation of large multi-cellular clusters with DCs, resulting in primary immune responses [65]. After intravenous antigen delivery, the first immune responses are documented in the BM and concomitantly in spleen, consistent with the accessibility of both sites to blood-borne antigens. Specifically, CD69 up-regulation was measured 4 h after challenge with ovalbumin, whereas the first cell division occurred 26 h later [65]. T-cell responses initiated in the BM gave rise to long-term immunological memory in mice lacking secondary lymphoid organs. 

## 3. Immunophenotypic and Functional Features of Leukemia Stem Cells (LSCs)

Leukemia cells and LSCs express antigens which are immunogenic and can be recognized by immune cells, as well as MHC molecules and costimulatory ligands that allow interaction with endogenous T cells [72,73]. Innate immune responses to leukemia have been clinically documented in patients [74]. The in vivo immunogenicity of leukemia-associated antigens (LAAs) has also been confirmed in patients receiving allogeneic hematopoietic stem cell transplantation (HSCT) for AML and CML. Cytotoxic T lymphocyte (CTL) responses have been reported against a broad range of LAAs and CTAs, including HOXA9 [75], proteinase-3 [75], survivin [76], WT1 [77] and preferentially expressed antigen in melanoma (PRAME) [78].

Some therapeutically-targetable leukemia antigens originate from oncogenesis itself and are leukemia cell-specific, such as the BCR/ABL1 fusion protein in CML and Philadelphia (Ph)+ ALL, PML/RAR-α in acute promyelocytic leukemia, FLT3-ITD and mutated NPM1 in AML and IDH1/2. However, few leukemia-specific chromosomal rearrangements give rise to antigenic proteins, and these include the fusion proteins AML1-ETO (t(8;21)), DEK-CAN (t(6;9)) and PML/RAR-α (t(15;17)).

The majority of antigens have been characterized as LAAs, i.e., molecules expressed on leukemic cells, but also on normal non-malignant cells. For example, WT1 is not a leukemia-specific molecule, being detected at low levels in various normal tissues, such as gonads, kidney and the hematopoietic system, but is highly over-expressed by leukemia cells. The expression of BMI-1, an epigenetic repressor of the CDKN2A tumor suppressor locus and regulator of human HSC self-renewal [17], is higher in CD34^+^ cells from patients with CML than in those from healthy donors, and also increases with disease progression from chronic to advanced phase [79]. Interestingly, BMI-1 can be a target of graft-versus-leukemia (GVL) responses in patients with CML receiving HSCT from an HLA-identical sibling [80]. High BMI-1 expression levels prior to allogeneic HSCT were an independent marker associated with lower incidence and severity of acute GVHD, lower non-relapse mortality and better OS in patients with chronic-phase CML. BMI-1-specific CTLs have been detected in 20% of HSC sibling donors and in 42% of HLA-A*0201 CML patients before HSCT [81]. The majority of BIM-1-specific CTLs released IFN-γ, but not IL-2, IL-4 or IL-10, upon exposure to the relevant peptide. In a minority of HSC donors, TNF-γ production and NK-cell degranulation were also detected. BMI-1-specific T cells exhibited a memory phenotype, promptly expanded in short-term 7-day cultures in vitro and could be detected in patients transplanted from CTL-positive HSC donors. Although the different failed to achieve statistical significance due to the small size of this CML cohort, patients whose donors showed immune responses to BMI-1 peptides experienced better LFS after HSCT (80%) compared with patients whose donors showed no BMI-1 immune response (60%) [81]. These studies this suggest that polycomb group proteins may have relevance for disease control by GVL responses and may be a potential biomarker to identify those patients likely to develop GVHD. The over-expression of BIM-1, CLL-1 and TIM-3 were also correlated with shorter OS in a cohort of 40 patients with newly diagnosed AML [82]. 

Other LAAs belong to the cancer testis antigen (CTA) family, a large group of immunogenic proteins that are normally expressed only in germ cells of the testes and, to a lesser extent, in ovaries and placental trophoblasts. Given the immune privileged status of the above tissues, CTAs are considered to be de facto tumor-specific antigens and are promising potential targets for tumor immunotherapy approaches. PRAME has been broadly characterized as an AML-associated CTA, although its expression pattern in normal tissues, such as the adrenal glands, the endometrium and the pancreas, is broader than that of “classical” CTAs. PRAME-specific T cells may also recognize normal kidney epithelial cells and dendritic cells [83].

Importantly, some LAAs might be down-regulated in LSCs compared with more differentiated leukemia cells. In one study, characterization of 5 AML samples via Affymetrix Hu133A microarrays allowed the identification of 261 DNA repair, signal transduction and cell cycle genes, the expression of which was significantly lower in AML-derived LSCs compared with CD34^+^CD38^+^ leukemia cells [84]. These findings were consistent with the increasing chromosomal aberrations and mutations that are typical of AML. Interestingly, CD123 (the trans-membrane α chain of the IL-3 receptor), a molecule previously identified as a LSC-specific marker in AML [85] and found to be co-expressed with CD33 in 70% of adult AML cases [5], was detected on LSCs, but not on bulk leukemia cells. CD123 was found to be expressed more frequently than CD33 or CD34 in a panel of primary human AML specimens [86]. Cell sorting experiments demonstrated that both CD123^dim^ and CD123^bright^ populations formed colonies in semisolid media and suggested that virtually all AML blasts are functionally CD123^+^. Importantly, expression of CD123 was no higher on phenotypically defined LSCs than on bulk leukemia cells.

The proportion of CD34^+^CD38^low/−^CD123^+^ cells has been shown to be highly variable in de novo AML, ranging from 0.01 to 67%, and to predict response to treatment and survival. Specifically, a frequency of CD34^+^CD38^low/−^CD123^+^ cells greater than 15% at diagnosis and an unfavorable karyotype correlated with lack of complete response to induction chemotherapy in 100 patients aged less than 65 years [87]. Also, a greater than 1% population of CD34^+^CD38^low/−^CD123^+^ cells negatively affected disease-free survival and OS within the intermediate and favorable karyotype groups.

The tumor necrosis factor (TNF) superfamily ligand-receptor pair CD70/CD27 has been shown to be expressed on AML blasts and AML stem/progenitor cells, but not on HSCs from healthy BM donors [88]. CD70/CD27 signaling activates stem cell gene expression programs and promotes cell proliferation in AML cells, and mediates drug resistance in CML [89]. Soluble CD27, the levels of which might reflect the extent of CD70/CD27 interactions in vivo, was significantly elevated in the sera of newly diagnosed AML patients and was a strong independent negative predictor of OS. Antibody blocking of CD70/CD27 interactions induced asymmetric cell divisions and differentiation in AML blasts and AML stem/progenitor cells, inhibited cell growth and colony formation, and significantly prolonged survival in murine AML xenografts. Interestingly, TKIs down-regulate micro-RNA miR-29 expression, leading to up-regulation of CD70. Combining TKIs with CD27/CD70 blockade can effectively eliminate human CD34^+^ CML stem/progenitor cells in xenografts and LSCs in a murine CML model, suggesting that CD70/CD27 interactions could be targeted to overcome treatment resistance in CML LSCs [89]. It has to be emphasized that TKI-resistant LSCs are extremely rare in the BM of patients with CML. In addition, LSCs cannot be selectively isolated from the normal HSCs that reconstitute the BM after TKI therapy.

CD96 (also referred to as T cell-activated increased late expression (TACTILE)) is a trans-membrane glycoprotein that mediates the adhesive interactions of activated T and NK cells during the late phase of the immune response. Alternative splicing generates multiple transcript variants encoding distinct isoforms [90]. CD96 is expressed on the majority of CD34^+^CD38^−^ AML cells in >60% of primary samples, in contrast with 5% of cells in normal, HSC-enriched populations [91]. When transplanted into irradiated newborn Rag2^−/−^γc^−/−^ mice, only CD96^+^ cells showed significant levels of engraftment in the BM of the recipient mice, suggesting that CD96 may serve as an LSC-specific therapeutic target. CD96 expression has been detected in AML patients with mutated WT1 and was associated with adverse clinical and biological features, such as higher white blood cell counts and percentage of blood blasts, and FLT3-ITD [92]. Biotinylated anti-CD96 antibodies (TH111) that efficiently deplete CD96-expressing LSCs could be employed for HSC graft engineering with magnetic cell sorting [93].

Interferon (IFN)-γ is a major effector cytokine secreted by CTLs. Murine LSCs and human CD34^+^ CML progenitor cells express receptors for IFN-γ [9]. Although CML LSCs express costimulatory molecules and MHC molecules and induce the proliferation of effector T cells in vitro, IFN-γ-stimulated, PD-L1/PD-L2-over-expressing LSCs have been shown to accelerate CML progression after serial transplantation in mice [9]. Adoptively transferred CTLs enhanced the expansion of LSCs via IFN-γ only in mice with high leukemia antigen load. When recipient mice were analyzed 18 h after transfer, an experimental setting where leukemia antigen load is low, neither LSC number nor IFN-γ serum levels were increased, and CTLs could successfully eradicate LSCs. Gene signatures indicative of IFN-γ responsiveness have been identified in human AML and CML cell lines [94]. Interestingly, higher expression levels of IFN-γ pathway genes, including IRF1, MX1, SOCS1, PD-L1 and IFNGR1, may correlate with worse clinical outcome in patients with AML [94,95], as backed by the analysis of patient survival using publicly available transcriptomic data from The Cancer Genome Atlas (TCGA) consortium (http://www.oncolnc.org/, access date: 20 December 2017).

IFN-γ is a prototypical inducer of indoleamine 2,3-dioxygenase-1 (IDO1) [96], which catabolizes the essential amino acid tryptophan to immune suppressive intermediates, collectively referred to as kynurenines, and is over-expressed by a variety of solid tumors and hematological malignancies, including 50% of patients with newly diagnosed AML [97,98,99,100,101]. Similarly, kynurenine levels are increased in approximately 45% of patients with untreated CML, correlate with tumor burden and normalize during the course of patient treatment [102]. An immunohistochemistry IDO1 score calculated by multiplying the grade of IDO1 staining intensity by the percentage of stained mononuclear cells was shown to predict induction failure and to negatively correlate with OS in adult patients with AML [103]. In vitro treatment of AML cell lines with IFN-γ translates into the up-regulation of functional IDO1 and other pro-inflammatory mediators, including cycloxygenase-2 [104]. Small molecule inhibitors of IDO1, i.e., indoximod (1-methyl-d-tryptophan) and epacadostat (INCB24360), are being tested in the clinic, with initial reports showing safety and tolerability in patients with advanced solid tumors [105,106,107,108]. Studies have suggested that IDO1 might be selectively elevated in tumor-initiating cells (TICs) from breast cancer, prostate cancer and mesothelioma cell lines, as well as primary human glioblastoma cells [109]. Serial transplantation of TICs was associated with IDO1 over-expression in recipient mice. All types of TICs also expressed higher levels of the tryptophan uptake machinery, including the LAT1 (SLC7A5)/CD98 (SLC3A2) heterodimeric amino acid transporter. It is presently unknown whether LSCs in AML and CML rely on IDO1 expression as an immune evasion strategy and whether patients with hematological malignancies may benefit from therapy with IDO1-targeting small-molecule inhibitors.

A phase 1b/randomized phase 2a clinical trial of indoximod in combination with idarubicin over three days and cytarabine over seven days (3 + 7) is actively recruiting patients with newly diagnosed AML aged >18 years with targeted completion in 2018 (clinicaltrials.gov identifier: NCT02835729). Results of the phase I portion of the clinical trial have shown that incorporation of indoximod into conventional remission induction and consolidation is well tolerated without adding significant toxicity and may improve clinical outcome [110]. As of 1 March 2017, five of six (83%) evaluable patients achieved MRD-negative CR after induction and remained MRD-negative after the first cycle of consolidation with high-dose cytarabine.

## 4. Targeting LSC-Associated Antigens to Overcome Therapeutic Resistance

**Figure 2:**
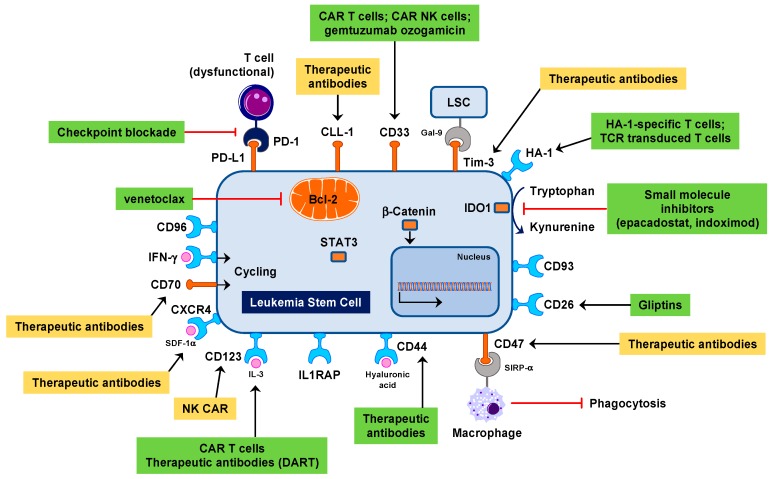
Actionable targets expressed in leukemia stem cells. Therapeutic strategies currently being evaluated include the use of chimeric antigen receptor (CAR)-modified T cells and antibodies such as bi-specific CD3 × CD123 dual affinity re-targeting (DART) molecules [117]. Green boxes highlight therapeutic strategies that are being investigated in clinical trials, such as CD33 [118] and CD44 targeting [119] and Bcl-2 antagonism [120], whereas yellow boxes denote therapeutic approaches, such as CXCR4 [21] and CD47 targeting [121] and TIM-3 blockade [8], that have been explored in murine models of leukemia. LSC = leukemia stem cell.

Allogeneic HSCT is an effective immunotherapy modality for patients with intermediate-risk and high-risk AML [111,112,113]. A review of records of 10,632 patients worldwide who were alive and disease free 2 years after receiving a myeloablative allogeneic HSCT for hematological malignancies before 2004 showed that the probability of being alive 10 years after HSCT is 84% for AMLs and 80% for MDSs [114]. However, life expectancy remained lower than anticipated due to non-relapse deaths mostly attributable to graft-versus-host disease (GVHD), infection, organ toxicity and second cancers. There is urgent need to develop more specific and less toxic approaches, especially for elderly patients. The identification of “actionable” immunotherapy targets within the LSC compartment would be highly beneficial to implement innovative approaches to clinical translation [73] (Figure 2). Strategies for targeting LSCs fall into two broad categories: therapies that eradicate LSCs (termed “LSC-specific”) and therapies that eradicate both the bulk of AML and the LSC compartment (termed “LSC-active”) [40]. The first defined LSC-specific immunophenotypic property was expression of CD123 within the CD34^+^CD38^−^ compartment [85]. Some of the differentially expressed molecules are being targeted in pre-clinical models of hematological malignancies and in clinical trials, mostly using antibody-based and cell-based therapeutic approaches (Figure 2). CD123 has been targeted with neutralizing monoclonal antibodies (e.g., 7G3) in NOD/SCID mice [115] and in patients with relapsed/refractory AML [116]. In one study, 7G3 treatment reduced the engraftment potential of AML-derived LSCs and improved mouse survival. 7G3 also inhibited IL-3-mediated intracellular signaling of isolated AML CD34^+^CD38^−^ cells in vitro and reduced their survival.

PTC-209 is a recently developed inhibitor of BMI-1. Pharmacological treatment of CML cells with PTC-209 has been shown to trigger cyclin G2 (CCNG2) expression, thus decreasing clonogenic activity [122]. Furthermore, BMI-1 and CCNG2 levels evolved inversely during CML progression, suggesting that BMI-1 could support acute transformation of CML through the silencing of a CCNG2-mediated tumor-suppressive autophagy response. Another small molecule inhibitor of BMI-1, PTC-596, triggers molecular events consistent with induction of p53-independent mitochondrial apoptosis in AML stem/progenitor cells, such as loss of membrane potential, conformational changes in Bax, cleavage of caspase-3 and externalization of phosphatidylserine [123]. PTC-596 also exhibited in vivo anti-leukemia activity in mice, while sparing normal HSCs. A phase 1, open-label, first-in-human, safety and pharmacokinetic study of PTC-596 is currently ongoing in patients with unresectable or metastatic solid tumors (ClinicalTrials.gov Identifier: NCT02404480). PTC-596 will be administered orally on a twice a week schedule for 4 weeks (one cycle). The objectives of the study will be to determine the recommended phase 2 dose and to establish preliminary proof of mechanism of action.

DT388IL-3 (SL-401) is a fusion protein containing the catalytic and translocation domains of diphtheria toxin fused to human IL-3 [124]. An inter-patient dose escalation trial in patients with chemo-refractory AMLs and MDSs has documented one complete response and 1 partial response in 40 evaluable patients with AML [124]. Of five MDS patient, one experienced a partial response. Toxicities were minimal and anti-DT388IL-3 antibodies developed in most patients between day 15 and 30. Other targeted therapeutics directed at CD123 include SL-401 and SL-501 [125,126]. SL-401 is a recombinant fusion protein composed of the catalytic and translocation domains of diphtheria toxin fused via a Met-His linker to IL-3. SL-401 has been administered to patients with CD123-expressing blastic plasmacytoid dendritic cell neoplasm (BPDCN), inducing several durable CR [125]. Intriguingly, both SL-401 and SL-501, a next-generation CD123-targeted therapy with increased binding affinity for the IL-3R and enhanced potency, inhibit the clonogenic growth and long-term colony formation potential and enhance imatinib-induced apoptosis of primary CML cells, including CML samples that harbored the T315I tyrosine kinase mutation [126]. In contrast, the additive effect of imatinib and either SL-401 or SL-501 on primary CML cells from patients with TKI-resistant disease were restricted to samples without the T315I mutation. A non-randomized multi-center clinical trial of SL-401 as consolidation therapy (Table 1) is ongoing in patients with adverse-risk AML in first CR following induction chemotherapy (clinicaltrial.gov Identifier: NCT02270463). A four-stage, non-randomized, open-label trial will explore the efficacy and safety of SL-401 in patients with AML and BPDCN (clinicaltrial.gov Identifier: NCT02113982). SL-501 also showed in vitro anti-tumor activity against a variety of cells lines from patients with Hodgkin and non-Hodgkin lymphoma, providing a rationale for further development of this therapeutic strategy [127].

SL-101 is an antibody conjugate comprising an anti-CD123 single-chain Fv fused to Pseudomonas exotoxin-A [128]. The anti-leukemia potency of SL-101 was initially measured in preclinical studies using a panel of AML cell lines. Colony-forming assays indicated that SL-101 selectively suppressed the function of leukemic progenitors, while sparing normal counterparts. Mechanisms underpinning the cytotoxic activity of SL-101 included rapid and efficient internalization of antibody, sustained inhibition of protein synthesis, induction of apoptosis, and blockade of IL-3-induced phosphorylation of STAT5 and AKT. In a PDX model of human AML engrafted in NSG mice, in vitro pre-treatment of LSCs with SL-101 impaired their repopulating capacity.

SGN-CD123A is an antibody-drug conjugate utilizing the pyrrolobenzodiazepine dimer linker and a humanized CD123 antibody with engineered cysteines for site-specific conjugation [129]. Mechanistically, SGN-CD123A induces activation of DNA damage response pathways, cell cycle changes, and apoptosis in AML cells. In vitro, SGN-CD123A mediated potent cytotoxicity of CD123^+^ AML cell lines and primary AML samples, including those from patients with unfavorable cytogenetic profiles or FLT3 mutations. In vivo, SGN-CD123A eradicated AML in a disseminated disease model, induced remission in a subcutaneous xenograft model, and significantly delayed growth in a multi-drug resistance xenograft model. Moreover, SGN-CD123A also resulted in durable complete leukemia remission in a AML PDX model. An ongoing dose-finding phase 1 clinical trial is evaluating the safety of SGN-123A in AML patients (NCT02848248).

CD123 may also be a viable target for AML-directed chimeric antigen receptor (CAR) T cell immunotherapy. Preclinical studies recently reported anti-AML activity of T cells transduced with CAR constructs containing a CD123-specific single-chain variable fragment in combination with CD28 or 4-1BB costimulatory domains and a CD3-ζ signaling domain [86,130]. In the first study by the City of Hope group, CD123-CAR-redirected T cells mediated potent effector activity against CD123^+^ cell lines and primary AML samples without eliminating granulocyte-macrophage and erythroid colony formation in vitro. Importantly, CD123 CAR T cells also exhibited anti-leukemia activity against a xenogeneic model of disseminated AML [130].

A second study by the Children’s Hospital of Philadelphia and University of Pennsylvania group showed that CD123 expression increases over time in vivo even in initially CD123^dim^ populations and that human T cells transduced with an anti-CD123-41BB-CD3ζ construct (CART123) could eradicate primary AML cells engrafted in immunodeficient mice, regardless of baseline CD123 expression [86,144]. However, a single administration of CART123 also ablated normal human HSCs, as predicted from the expression of CD123 on normal circulating B cells, myeloid cells and megakaryocytes. Also, phenotypically defined human stem/progenitor cells were undetectable in CART123-treated animals at one month post-treatment, correlating with the known expression of CD123 on progenitor cells.

Severe hematological toxicity of CD123-redirected CAR T cells could be obviated for by CAR T-cell depletion with optimal timing after AML eradication [144]. Three CAR T-cell termination strategies were recently evaluated in a follow-up study, including the use of transiently active anti-CD123 mRNA CART (RNA-CART123), T-cell ablation with alemtuzumab after treatment with anti-CD123-41BB-CD3ζ T cells (CART123), and T-cell ablation with rituximab after treatment with CD20-coexpressing CART123 (CART123-CD20) [144]. Rapid and durable leukemia elimination in murine xenograft models of human AML could be consistently detected and required CAR T-cell persistence for 4 weeks prior to ablation. Importantly, subsequent antibody-mediated depletion of CART123 or CART123-CD20 did not impair leukemia remission. These studies will facilitate the clinical implementation of T-cell depletion strategies to augment the feasibility of CAR T-cell therapies for patients with AML. Primary human NK cells can be isolated, expanded and transduced to express CARs against CD123 using the good manufacturing practice (GMP)-compliant Prodigy™ device (Miltenyi Biotech, Germany) [136]. Compared with freshly isolated NK cells, CAR-engineered NK cells expressed higher levels of NKp30, NKp44, NKG2D and TRAIL molecules, secreted high levels of IFN-γ and efficiently lysed CD123-expressing targets in vitro. Clinical trials are ongoing to evaluate the potential benefit of infusing CAR-NK cells in patients with CD7^+^ AML (clinicaltrials.gov Identifier: NCT02742727) and CD33 or CD123-expressing AML (Table 1).

Flotetuzumab (MGD006/S80880), a T-cell redirecting (CD123 × CD3) bispecific dual antigen retargeting antibody (DART), has been tested in adults with relapsed/refractory AMLs and MDSs via a phase 1 study (NCT01766375) [117]. In a preliminary report of data from 45 patients, toxicity with drug-related adverse events ≥G3 (primarily infusion-related reactions/cytokine release syndrome) were observed in 44% of patients, but were manageable with supportive care. Anti-leukemia activity of flotetuzumab was documented in 57% of patients, and the overall response rate was 43% with greater responses observed in patients treated at ≥500 ng/kg/day dosing. Markers of T-cell activation, including CD25, CD69 and PD1, were detected in the peripheral blood of patients after treatment [134,145].

The myeloid differentiation antigen CD33 is expressed on leukemic blasts in 85% to 90% of AML patients [118]. Gemtuzumab ozogamicin (GO) utilizes an anti-CD33 antibody conjugated to the anti-tumor antibiotic calicheamicin. GO has potent in vitro cytotoxicity against human AML cell lines [146,147,148,149] and has demonstrated clinical activity in both adults and children with AML [131,133,150,151]. Based on these favorable responses, GO was approved in 2017 by the U.S. FDA for use in adults with newly diagnosed CD33^+^ AML and in patients aged 2 years and older with relapsed/refractory CD33^+^ AML. Levels of CD33 surface expression may also correlate with clinical responses to GO [152], and a recent study also demonstrates potential importance of CD33 splice variants for GO response [153]. Interestingly, the CD33 single nucleotide polymorphism rs12459419 C>T in the splice enhancer region was noted to eliminate the CD33 immunoglobulin variable domain, which is the antibody-binding site for GO. Results of a recent Children’s Oncology Group randomized clinical trial of GO in children with newly diagnosed AML suggest that patients with the CC genotype for rs12459419 have a substantial response to GO, an observation which points to CD33 polymorphisms as potential biomarkers for the selection of patients with greatest likelihood of significant response to immunotherapy with GO [153]. Studies to date have not determined whether GO, besides acting on the more mature CD33^+^ progeny, can indeed directly kill CD33^+^ LSCs in vivo, and whether long-term benefit from GO is related to successful targeting of LSCs, including AML cases that harbor CD33^−^ LSCs. It has been proposed that CD33^−^ LSCs remaining after chemotherapy-induced bulk reduction may enter cell cycle, acquire CD33 and eventually become susceptible to CD33-targeting drugs [118].

CD33-specific CAR T cells (CART33) are actively being developed with the anti-CD33 single chain variable fragment used in gemtuzumab ozogamicin (clone My96) [154]. CART33 exhibited effector functions in vitro, eradicated leukemia and prolonged survival in AML xenografts. Importantly, CART33 also induced human lineage cytopenia and reduced myeloid progenitors in xenograft models of hematopoietic toxicity, suggesting that permanently expressed CD33-specific CART cells would have unacceptable toxicity if infused into patients with AML [154]. When a transiently expressed mRNA anti-CD33 CAR was designed, potent but self-limited activity was detected, indicating that this modification could be pursued further to avoid long-term myelosuppression in patients with AML. Another approach focuses on the use of second-generation anti-CD33 CARs that incorporate a 4-1BB-CD3ζ signaling tail previously shown to be effective in clinical trials of both chronic and acute lymphocytic leukemia [155]. Anti-CD33 CAR T cells exhibited anti-leukemia effects in NSG mice and killed primary CD33-expressing AML cells.

C-type lectin-like molecule 1 (CLL-1) was identified by a Dutch group of investigators as an AML LSC-specific surface molecule [156]. CLL-1 expression is prevalent in AML, both at diagnosis and relapse, and is not expressed on HSCs in normal and regenerating BM samples [156]. The CD34^+^CLL-1^+^ population, containing the CD34^+^CD38^−^CLL-1^+^ cells, does engraft in NOD/SCID mice with outgrowth to CLL-1^+^ blasts. A high CLL-1^+^ fraction was associated with quick relapse. Bispecific antibodies that redirect the cytotoxic activity of effector T cells by binding to CD3, the signaling component of the T-cell receptor, and a tumor target such as CD19 on ALL show encouraging clinical results [157,158]. The safety and potency of target cell depletion of a CD3 T cell-dependent bispecific full-length human IgG1 therapeutic antibody targeting CLL-1 has been recently reported [141]. CLL-1 CAR T cells have also been engineered to express inducible capspase-9, a safety ‘suicide switch’ that could accelerate the clinical development of this immunotherapy strategy by allowing control of unwanted T-cell reactivity against normal myeloid cells [142].

CD47 is a broadly expressed transmembrane protein that serves as the ligand for signal regulatory protein α (SIRPα), which is expressed on phagocytic cells including macrophages and DCs. When activated, SIRPα initiates a signal transduction cascade resulting in inhibition of phagocytosis. CD47 is preferentially expressed on AML-derived LSCs cells compared to their normal counterpart and inhibits their phagocytosis through the interaction with an inhibitory receptor on phagocytes [121]. Lower CD47 expression levels have been reported in AML patients with t(8;21) compared with patients with unfavorable cytogenetic features such as FLT3-ITD. Moreover, CD47 expression predicted worse OS in three independent cohorts of adult AML patients dichotomously stratified into CD47^low^ and CD47^high^ expression groups. Treatment of mice engrafted with human LSCs with therapeutic anti-CD47 antibodies resulted into AML depletion and targeting of LSCs. Studies that used SIRPα-Fc fusion protein to disrupt SIRPα-CD47 engagement have suggested that macrophage-mediated phagocytosis and clearance of AML stem cells depend on absent SIRPα signaling [159]. Importantly, SIRPα-Fc treatment did not significantly enhance phagocytosis of normal hematopoietic targets by activated human macrophages. The potential of CD47 to serve as an antibody target could theoretically be hindered by its low-level expression in many tissues. However, rat anti-mouse CD47 antibodies have shown safety and lack of significant toxicity when administered to mice, with the only exception of severe neutropenia [121]. Different monoclonal antibodies targeting CD47 are being tested in patients with advanced AML and MDSs (clinicaltrials.gov Identifiers: NCT02678338, NCT02367196).

CD44 is a type I transmembrane protein and functions as the major cellular adhesion molecule for hyaluronic acid, a component of the extracellular matrix. CD44 is expressed in most human cell types and has been implicated in myeloid leukemia pathogenesis. A naturally occurring leukemogenic splice variant of t(8;21), *AML1-ETO9a*, significantly increases the expression of CD44 at both RNA and protein levels [160]. Furthermore, the CD44 promoter is bound by *AML1-ETO9a* and *AML1-ETO* at the chromatin level, indicating that CD44 expression links the 8;21 translocation to the regulation of a cell adhesion molecule that controls AML growth. Ligation of CD44 with activating antibodies (H90) eradicates AML LSCs in NOD-SCID mice by blocking LSC trafficking to supportive microenvironments and by altering their stem cell fate [6]. In vitro H90 treatment led to multiple changes indicative of terminal differentiation, such as increased expression of lineage antigens, ability to reduce nitroblue tetrazolium and acquisition of mature morphology. The number of CD34^+^CD38^−^ cells within the AML graft in both BM and peripheral blood was considerably reduced in H90-treated mice as compared with control mice [6]. RG7356, a recombinant anti-CD44 IgG1 humanized monoclonal antibody, has been administered to 44 patients with refractory/relapsed AML or patients not eligible for intensive chemotherapy in a phase I dose-escalation study [119]. Two patients achieved complete response with incomplete platelet recovery or partial response, respectively. One patient had stable disease with hematologic improvement. Overall, RG7356 was safe and well tolerated with one dose-limiting toxicity (grade 3 hemolysis exacerbation) occurring after one 1200 mg dose. Whereas the majority of adverse events were mild or moderate, infusion-related reactions occurred in approximately 60% of AML patients, mainly during cycle 1. Two patients experienced grade 3 drug-induced aseptic meningitis. Based on the results of this study, the recommended dose for future AML evaluations will be 2400 mg every other week. Other approaches to target CD44-expressing LSCs include the manufacturing of CAR T cells redirected against the CD44 isoform variant 6 (CD44v6) and containing a CD28 signaling domain [143]. CD44v6 CAR T cells required in vitro activation with cytokines, such as IL-7 and IL-15, for anti-tumor efficacy in vivo and spared normal HSCs and CD44v6-expressing normal keratinocytes when administered to AML-bearing mice. The co-expression of a suicide gene allowed rapid, efficient ablation of CD44v6 CAR T cells following pharmacologic ablation and rescued mice from acute graft-versus-host disease.

TIM-3 is a type 1 cell-surface glycoprotein originally identified in murine CD4^+^ Th1 cells. In humans, TIM-3 is expressed also in a fraction of T cells, NK cells, monocytes, and DCs. TIM-3 is broadly expressed in human AML, with the only exception of acute promyelocytic leukemia and is not detected in normal HSCs [8]. TIM-3^+^, but not TIM-3^−^, AML cells were shown to reconstitute human AML in immunodeficient mice, suggesting that the TIM-3^+^ leukemic population contains most functional LSCs. Moreover, anti-human TIM-3 mouse IgG2a antibodies with complement-dependent and antibody-dependent cellular cytotoxic activities inhibited the engraftment of AML after xenotransplantation and, when administered directly to mice grafted with human AML, they eliminated LSCs capable of reconstituting human AML in secondary recipients [8]. Anti-TIM-3 monoclonal antibodies (TSR-022, MBG453 and LY3321367) are being tested in patients with advanced solid tumors either as monotherapy (clinicaltrial.gov Identifier: NCT02817633) or in combination with PD1 blockade (clinicaltrial.gov Identifier: NCT02608268) or PD-L1 blockade (clinicaltrial.gov Identifier: NCT03099109).

## 5. Conclusions

The original conceptual framework that AML development recapitulates the normal hematopoietic hierarchy might represent an oversimplification of the complex biology of AML. LSCs may in fact reside in more than one population, and their functional heterogeneity and remarkable plasticity are increasingly being recognized.

Inflammatory cytokines such as IFN-γ and signaling via CD27 might induce the expansion of LSCs. A deeper understanding of the immune BM niche will further support and inform the development of immunotherapies targeting LSCs. Monoclonal antibodies and T-cell-based approaches targeting candidate LSC-specific molecules are being developed in the clinic with encouraging results. It is conceivable that LSC-directed therapies will have to be offered in combination with conventional treatments, either before or concurrent with chemotherapy, in order to avoid chemotherapy-induced evolution and increased complexities of the LSC population [15,40]. Recent studies have shown a 10- to 100-fold increase in LSC frequency at relapse using paired specimens from AML patients at diagnosis and following relapse after conventional chemotherapy [15]. The therapeutic potential of LSC-directed therapies is also being explored in the post-allogeneic HSCT setting for AML patients at high risk of relapse. For example, the administration of PF-04449913, a small molecule inhibitor of the hedgehog pathway, is being pursued in AML patients beginning on post-transplantation day 80 with the aim to inhibit aberrant hedgehog signalling and LSC survival and expansion in AML (clinicaltrials.gov Identifier: 01841333).

Our improved understanding of the biology of LSCs will allow for more specific targeting of the LSC population with curative intent [161]. Issues that need to be addressed include the development of analytical tools to identify and quantify LSCs in patient samples, before and after treatment, including immunophenotypic reagents and techniques [93]. Principles and considerations for the design of novel LSC-targeting therapies are currently being defined [161].

New endpoints to evaluate response are likely to be required to assess LSC-directed therapies, i.e., patient survival rather than response rates, which might not be increased by therapies that target a tiny proportion of the bulk disease [40]. Although identifying and pursuing antigenic targets to eradicate LSCs is an active area of research, the efficacy of this approach is still unknown and may be limited by the relative plasticity of LSC phenotypes. Many things remain unknown, but preclinical and clinical studies to date nonetheless suggest that LSC-targeting therapies may ultimately have therapeutic promise for patients with hematological malignancies.

## Figures and Tables

**Table 1:** Completed and ongoing clinical trials targeting antigens expressed on LSCs.

Disease	Target on LSCs	Strategy/Approach	Reference(s)/clinicaltrial.gov Identifier
AML	CD33	Gemtuzumab ozogamicin; transduced autologous T cells; CAR-NK cells	[131,132,133]; NCT02944162; NCT03126864
AML; CML; BPDCN	CD123	Monoclonal antibodies (CSL360; CSL362); immunotoxins (DT388IL-3 (SL-401); SL-501); antibody-drug conjugates (SGN-CD123A); DART molecules (flotetuzumab); CAR T cells (CD123CAR-41BB-CD3z-EGFRt; UCART123); CAR-NK cells	[116,117,124,129,134,135,136]; NCT03114670; NCT01632852; NCT03190278; NCT02159495; NCT02270463; NCT02113982
AML	Bcl-2	Bcl-2 inhibitor (venetoclax)	[120,137]
AML, MDS, ALL	WT1	CAR T cells	NCT02550535; NCT01621724; NCT01266083
AML relapsing or at risk of relapse after HSCT	HA-1	HA-1-specific T cells; CD4^+^ and CD8^+^ T cells transduced with a lentiviral vector incorporating the HA-1 TCR transgene construct	NCT03326921; [138,139,140]
CML	BMI-1	Pharmacological inhibition (PTC-209 and PTC-596)	NCT02404480; [122,123]
AML	CLL-1	Bi-specific antibodies; CAR T cells	[141,142]
AML	CD44; CD44v6	Monoclonal antibodies (RG7356); CAR T cells (CD44v6.CAR28z^+^)	[119,143]
AML, MDS, lymphoma	CD47	Monoclonal antibodies (Hu5F9-G4; CC-90002; TTI-621 (SIRPαFc))	NCT02678338; NCT02367196; NCT02663518

DART = Dual Affinity Re-Targeting antibody; WT1 = Wilms Tumor 1; HA-1 = Minor histocompatibility antigen 1; CAR = Chimeric Antigen Receptor; UCAR = Universal Chimeric Antigen Receptor; ALL = Acute Lymphoblastic Leukemia; AML = Acute Myeloid Leukemia; MDS = Myelodysplastic Syndrome; CML = Chronic Myeloid Leukemia; BPDCN = Blastic Plasmacytoid Dendritic Cell Neoplasm.
